# Nightmares affect the experience of sleep quality but not sleep architecture: an ambulatory polysomnographic study

**DOI:** 10.1186/s40479-014-0023-4

**Published:** 2015-02-13

**Authors:** Franc Paul, Michael Schredl, Georg W Alpers

**Affiliations:** Central Institute of Mental Health, Medical Faculty Mannheim/Heidelberg University, J5, Mannheim, 68159 Germany; University of Mannheim, School of Social Sciences, Department of Psychology, Clinical and Biological Psychology and Psychotherapy, L13, 15-17, Mannheim, 68131 Germany

**Keywords:** Nightmare, Dream, Sleep quality, Ambulatory sleep recording

## Abstract

**Background:**

Nightmares and bad dreams are common in people with emotional disturbances. For example, nightmares are a core symptom in posttraumatic stress disorder and about 50% of borderline personality disorder patients suffer from frequent nightmares. Independent of mental disorders, nightmares are often associated with sleep problems such as prolonged sleep latencies, poorer sleep quality, and daytime sleepiness. It has not been well documented whether this is reflected in objectively quantifiable physiological indices of sleep quality.

**Methods:**

Questionnaires regarding subjective sleep quality and ambulatory polysomnographic recordings of objective sleep parameters were collected during three consecutive nights in 17 individuals with frequent nightmares (NM) and 17 healthy control participants (HC).

**Results:**

NM participants reported worse sleep quality, more waking problems and more severe insomnia compared to HC group. However, sleep measures obtained by ambulatory polysomnographic recordings revealed no group differences in (a) overall sleep architecture, (b) sleep cycle duration as well as REM density and REM duration in each cycle and (c) sleep architecture when only nights with nightmares were analyzed.

**Conclusions:**

Our findings support the observation that nightmares result in significant impairment which is independent from disturbed sleep architecture. Thus, these specific problems require specific attention and appropriate treatment.

## Background

Nightmares are commonly defined as disturbing dreams characterized by awakening from (REM) sleep with very vivid and detailed dream recall. Dream content is typically related to threats to survival, security or self-esteem [1,2]. For the diagnosis of nightmare disorder, dream content and the awakening must cause clinically significant distress or impairment in important areas of functioning [2,3]. At least one nightmare per week is often used as criterion as this frequency seems to cause clinically significant distress [4]. Nightmares are associated with disturbed sleep, low well-being and affect daytime mood and behavior. Nightmare disorder is a very common comorbidity in nearly all psychiatric conditions. In borderline personality disorder (BPD), for example, up to 50% are troubled by frequent nightmares [5-7]. Correspondingly, BPD can be found in nearly one quarter of people who suffer from nightmares [8]. Moreover, nightmares are also a core symptom in posttraumatic stress disorder (PTSD) with about two thirds of patients suffering from terrifying dreams [9].

Anxiety or fear are often reported as the predominant emotions of nightmares, but recent research suggests that other, less intense emotions like frustration or guilt can be involved as well [10]. The prevalence of frequent nightmares in the general population is about 5% in adults [11-13] and even higher in childhood and adolescence [14-18].

To compare results of different studies, it is important to define nightmares and distinguish them from other (unpleasant) dreams. A common criterion is that nightmares are very unpleasant and disturbing dreams which *awaken* the dreamer [3,10], compared to other unpleasant and disturbing dreams that do *not awaken* a person [10]. The distinction is also important because results indicate that there is a stronger relationship between impaired well-being and nightmare frequency than bad dream frequency [19]. Furthermore, the same study found that people with frequent nightmares also reported to have frequent bad dreams, whereas those with a high frequency of bad dreams did not experience frequent nightmares [19].

People who frequently experience nightmares often report massively reduced sleep quality. Nightmare sufferers (between 10–16 nightmares on average per month) scored worse on self-report measures; they fear going to sleep, experience awakenings from sleep, have difficulty falling asleep again and have fitful sleep patterns [20]. About 50-60% with much less frequent nightmares (2.7 ± 3.3 in the last month) also reported difficulties to fall asleep again and described their post nightmare sleep as more restless and uncomfortable as well as less refreshing [21]. Similar results were found in a large sample of university students; nightmare frequency correlated significantly with low sleep quality scores (e. g., feeling refreshed after sleep, having headaches in the morning or fitful sleep) [22].

Furthermore, research has shown that nightmares affect daytime functioning in terms of reducing general well-being, increasing anxiety before sleep and after nightmares and correspond with negative mood and higher stress before and after sleep [19,21,23-25]. Although there is a correlation between nightmare frequency and waking anxiety in some studies [19,21,25], other research suggests that nightmare frequency is independent from general waking anxiety measures [4,26,27]. One explanation could be that it is not nightmare frequency per se, but nightmare distress which influences psychopathology, e. g., anxiety or depression symptoms [4,25,28,29]. Nightmare distress describes the suffering and waking effects of having frequent nightmares that is determined by the way in which people deal with their dreams and how they influence their thoughts and behavior [3]. Frequent nightmares and nightmare distress were found to be strongly associated with neuroticism, irrespective of mental disorders [21,30].

There are only few polysomnographic studies which evaluated sleep architecture and their results are conflicting: Some studies found objective sleep disturbances [31,32] in frequent nightmare sufferers, others did not [33,34]. The two positive studies [31,32] observed more periodic leg movements, increased wakefulness after sleep onset, relatively less non-REM and slow wave sleep and a higher relative REM duration in frequent nightmare sufferers.

There are clearly disadvantages when dreams or nightmares are investigated in laboratory settings: People tend to dream about the laboratory environment and those dreams are usually less emotional and shorter than at home [35-38]. In related disciplines, it has been well recognized that critical events related to psychopathology – such as panic attacks – require assessments outside of the laboratory, when and where those events occur [39].

Therefore, the present study investigates nightmares in a natural sleep environment and assesses both self-report and psychophysiological indicators of sleep disturbances. It was expected that participants with frequent nightmares experience poorer sleep quality compared to those with normal sleep and that this can be confirmed by ambulatory polysomnographic assessments.

## Methods

### Participants

We present results from 34 participants, 17 individuals with frequent nightmares (DSM criteria [2] with a minimum frequency of at least once a week; mean age 25.18 ± 3.66 yrs; 16 female, 1 male) and 17 healthy control participants (mean age 24.31 ± 4.69 yrs; 16 female, 1 male). The groups did not differ in age (*t* = 0.590, p = 0.563) and depression scores (*t* = −0.898, p = 0.377). Exclusion criteria were the diagnosis of other mental and sleep disorders, medical conditions or medication with influences on mood, sleep, or the HPA axis (including antidepressants), current drug or alcohol abuse and shift work. Telephone interviews were conducted to screen participants. SCID [40] questions were applied to assess mental disorders. Only one participant showed subclinical signs of a prior episode of major depression and was therefore included in the study. All other participants did not reveal any signs of prior or current mental conditions. Furthermore, no drugs other than contraceptives were taken. Nightmares were defined as strong negative dreams that awaken the dreamer and were distinguished from bad dreams without awakening. All participants signed informed consent and the study was approved by the local ethics committee.

### Sleep recordings

Ambulatory sleep recordings were performed with a V-Amp amplifier (BrainProducts GmbH, Gilching, Germany). Raw data was sampled at 500 Hz and processed and filtered for sleep scoring according to the AASM manual [41]. Ag/AgCl electrodes were applied and prior to attachment, skin was pretreated with abrasive paste. Sleep EEG was measured by ambulatory polysomnographic recording (PSG) (F4-A1, C4-A1, O2-A1, F3-A2, C3-A2, O1-A2). In addition, we recorded horizontal and vertical eye movements (EOG), submental and leg electromyogram (EMG; left and right anterior tibial muscles), as well as electrocardiogram (ECG). Periodic leg movements were only measured in the first night. Sleep was visually scored according to standard criteria of the AASM [41]. Sleep measures included sleep period time (SPT; in minutes), sleep efficiency index (SEI, %), sleep onset latency (in minutes), REM sleep latency (in minutes), REM sleep density (%), number of nocturnal awakenings, number of REM periods, arousal index (AI), amount of wakefulness after sleep onset (W%), amount of light sleep (N1%), amount of normal sleep (N2%), amount of slow wave sleep (N3%) and amount of REM sleep (R%) as well as arousal index in REM sleep (AIR).

### Questionnaires

Subjective sleep quality as well as depressiveness was assessed by different questionnaires including the Landecker Inventar zur Erfassung von Schlafstörungen LISST [42], Schlaffragebogen-A (SF-A) and Schlaffragebogen-B (SF-B) [43], Epworth Sleepiness Scale (ESS) [44] and Beck Depression Inventory (BDI) [45], all German versions. The SF-B questionnaire measures composite scores including sleep quality, feeling of being refreshed in the morning, feeling balanced/relaxed in the evening, feeling exhausted in the evening and psychosomatic symptoms in the sleeping period over the last two weeks. The SF-A yields the same composite scores prospectively for each night the questionnaire was completed. The LISST results in composite scores to detect different symptom classes of sleep disorders including problems with sleep/wake rhythm, insomnia symptoms, sleep quality, parasomnias and tiredness during the day. Nightmare frequency was measured on an eight-point rating scale [46] (‘How often do you experience nightmares?’ 0 = never, 1 = less than once a month, 2 = about once a month, 3 = twice or three times a month, 4 = about once a week, 5 = several times a week and 6 = almost every morning). In order to obtain mornings with dream recall per week, the scale was recoded using the class means (0 → 0, 1 → 0.125, 2 → 0.25, 3 → 0.625, 4 → 1.0, 5 → 3.5, 6 → 6.5).

### Procedure

Each participant slept three consecutive nights in their normal sleep environment at home for polysomnographic recordings (PSG). After all electrodes were attached, sleep laboratory staff left and participants went to bed at their regular times. Participants had to write dream reports on each nocturnal awakening with dream recall (results will be reported elsewhere). After awakening in the morning, the SF-A questionnaire was completed. Again, a member of the sleep laboratory detached the electrodes and controlled recordings. The other questionnaires were handed out after the last night to ensure that participants complete them later and not include the three study nights in their assessments.

### Hypothesis

We expect that people with frequent nightmares show worse subjective and objective sleep quality. Regarding the ambulatory polysomnographic recordings, we anticipate that people with frequent nightmares have higher arousal indices and more awakenings than control participants. No other expectations were made because this is the first study up to date that examines the sleep of nightmare sufferers in their home environment. On an exploratory level we want to investigate whether the sleep architecture differs between participants with frequent nightmares and healthy controls.

### Statistical analysis

Group comparisons were computed by independent *t*-tests and repeated measures analysis of variance with nights as within-subject factor to account for multiple measures within one participant. The significance level was set as alpha = 0.05. All group comparisons for the subjective sleep measures are one-tailed unless otherwise mentioned. Group comparisons of PSG results were two-tailed except AI, AIR and number of awakenings because we expected those to be higher in NM participants. Effect sizes are presented as d (d = 0.20: small effect; d = 0.50: medium effect; d = 0.80: large effect) or η_p_^2^.

## Results

### Dream recall

Overall, 96 dreams were reported in 102 nights of ambulatory polysomnographic recording. NM participants provided 49 dream reports, control subjects the remaining 47. Eleven participants in the NM group reported 13 nightmares (two participants with two nightmares), five in the first night, three in the second night and five in the third night. Also one HC participant reported a nightmare in the first night.

### Retrospective sleep questionnaires

Results of the retrospective sleep questionnaires are presented in Table 1. NM subjects scored worse on nearly all subjective sleep measures. Mean nightmare frequency in the NM group was ‘several times a week’ whereas HC participants reported to have nightmares between ‘less than once a month’ and ‘once a month’ (HC: 0.27 ± 0.30; NM: 3.08 ± 2.13; *t* = −5.040, p < 0.001, d = 1.91). Sleep quality was assessed by two questionnaires separately. Both measures revealed lower values in NM subjects (LISST: *t* = −2.864, p = 0.004, d = 1.03; SF-B: *t* = −3.903, p < 0.001, d = 0.92). Regarding other sleep complaints, NM participants scored significantly higher in insomnia problems (*t* = −3.697, p < 0.001, d = 1.33) as well as parasomnia complaints (*t* = −3.903, p < 0.001, d = 1.40) and marginally higher in problems with sleep/wake pattern (*t* = −1.615, p = 0.059, d = 0.58). No group differences were found in apnea symptoms (*t* = −1.528, p = 0.145, d = 0.57; two-tailed), restless legs symptoms (*t* = 0.381, p = 0.706, d = 0.13; two-tailed) and psychosomatic symptoms in the sleeping period (*t* = −1.112, p = 0.139, d = 0.40). Concerning daytime dysfunction, nightmare participants revealed significantly worse scores in daytime sleepiness (*t* = −1.596, p = 0.03, d = 0.70), daytime tiredness (*t* = −2.353, p = 0.013, d = 0.86), feeling of being refreshed after sleep (*t* = 1.713, p = 0.049, d = 0.62) and feeling balanced/relaxed in the evening (*t* = 2.911, t = 0.004, d = 1.05). No difference was found for feeling exhausted in the evening (*t* = 0.406, p = 0.344, d = 0.40).

**Table 1:** **Sleep questionnaire results comparing frequent nightmare sufferers (NM) with healthy controls (HC)**

**Scale (questionnaire)**	**NM**	**HC**	**NM vs. HC**		**Effect size**
			***t***	**p**	**d**
Daytime sleepiness (ESS)	9.53 ± 3.27	7.44 ± 2.68	−1.596	0.03*	0.70
Sleep quality (LISST)	21.04 ± 5.34	15.13 ± 6.10	−2.864	0.004*	1.03
Apnea symptoms (LISST)	5.40 ± 2.59	4.33 ± 0.82	−1.528	0.145	0.57
RLS symptoms (LISST)	7.67 ± 2.06	8.06 ± 3.49	0.381	0.706	0.13
Problems with sleep/wake pattern (LISST)	19.20 ± 6.33	15.69 ± 5.78	−1.615	0.059*	0.58
Insomnia symptoms (LISST)	17.87 ± 4.93	11.75 ± 4.28	−3.697	< 0.001*	1.33
Tiredness during the day (LISST)	15.47 ± 6.63	10.75 ± 4.17	−2.353	0.013*	0.86
Parasomnias (LISST)	18.68 ± 6.60	10.69 ± 4.70	−3.903	< 0.001*	1.40
Sleep quality (SF-B)	3.39 ± 0.59	3.88 ± 0.47	2.603	0.007*	0.92
Feeling of being refreshed (SF-B)	2.59 ± 0.77	3.09 ± 0.83	1.713	0.049*	0.62
Feeling balanced/relaxed in the evening (SF-B)	3.02 ± 0.59	3.69 ± 0.68	2.911	0.004*	1.05
Feeling exhausted in the evening (SF-B)	3.84 ± 0.59	3.94 ± 0.68	0.406	0.344*	0.16
Psychosomatic symptoms while sleeping (SF-B)	1.90 ± 0.73	1.66 ± 0.45	−1.112	0.139*	0.40

ESS: Epworth Sleepiness Scale [41], LISST: Landecker Inventar zur Erfassung von Schlafstörungen [39], SF-B: Schlaffragebogen-B [40].

Results are presented as mean ± standard deviation; effect sizes as d.

*One-tailed t-test.

### Subjective sleep measures in the study nights

Two participants provided not enough valid answers in the sleep questionnaire regarding subjective sleep quality of each night of the study so results are presented for 32 subjects (see Table 2). Analysis revealed that sleep quality increased over the three nights (*F*(2,60) = 20.706, p < 0.001, η_p_^2^ = 0.408) in both groups. A significant group effect (*F*(1,30) = 2.599, p = 0.04, η_p_^2^ = 0.079) indicated that NM participants generally scored worse in sleep quality. NM subjects felt less refreshed after sleep indicated by a significant group effect (*F*(1,30) = 7.678, p = 0.005, η_p_^2^ = 0.204). Moreover, the NM group felt less balanced in the evening (*F*(1,30) = 6.330, p = 0.009, η_p_^2^ = 0.174). No group or main effect was found for feeling exhausted in the evening (*F*(1,30) = 0.242, p = 0.313; *F*(2,60) = 0.476, p = 0.312, resp.). Psychosomatic symptoms in the sleeping period generally decreased from N1 to N3, indicated by a significant main effect of Night (*F*(2,60) = 3.310, p = 0.044, η_p_^2^ = 0.099); there was no difference between the groups (*F*(1,30) = 0.002, p = 0.485).

**Table 2:** **Self reported sleep measures of all study nights collected by SF-A questionnaire for nightmare sufferers (NM) and healthy controls (HC)**

**Measure**	**Night 1**		**Night 2**		**Night 3**	
	**NM**	**HC**	**NM**	**HC**	**NM**	**HC**
Sleep quality	2.63 ± 0.64	3.03 ± 0.81	3.44 ± 0.58	3.49 ± 0.69	3.33 ± 0.60	3.75 ± 0.51
Feeling of being refreshed after sleep	2.49 ± 0.59	3.13 ± 0.83	2.85 ± 0.72	3.20 ± 0.84	2.82 ± 0.61	3.40 ± 0.78
Feeling balanced/relaxed in the evening	3.19 ± 0.70	3.63 ± 0.65	3.36 ± 0.78	3.73 ± 0.57	3.29 ± 0.58	3.93 ± 0.60
Feeling exhausted in the evening	2.98 ± 0.72	2.81 ± 0.78	2.94 ± 0.56	2.94 ± 68	3.06 ± 0.54	2.91 ± 0.74
Psychosomatic symptoms while sleeping	1.79 ± 0.52	1.76 ± 0.49	1.67 ± 0.65	1.68 ± 0.47	1.49 ± 0.47	1.60 ± 0.43

SF-A: Schlaffragebogen-A [40].

Results are presented as mean ± standard deviation.

### Ambulatory polysomnography

Results from the ambulatory polysomnographic recordings (see Table 3) revealed no difference in SPT between the groups (*F*(1,32) = 1.002, p = 0.324). SEI generally increased over the three nights (*F*(2,64) = 12.845, p < 0.001, η_p_^2^ = 0.286) but did not differ between groups (*F*(1,32) = 0.062, p = 0.805). In all participants the amount of time awake decreased indicated by a significant Night effect for W% (*F*(2,64) = 13.595, p < 0.001, η_p_^2^ = 0.298) while REM% increased (*F*(2,64) = 3.351, p = 0.041, η_p_^2^ = 0.095). No group differences could be found for both measures (*F*(1,32) = 0.088, p = 0.768; *F*(1,32) = 0.021, p = 0.884). There was a significant Night effect for the number of REM periods (*F*(2,64) = 5.557, p = 0.006, η_p_^2^ = 0.148). Group comparisons for the amount of awakenings, AI and AIR were not significant (*F*(1,32) = 0.005, p = 0.942; *F*(1,32) = 1.389, p = 0.247; *F*(1,32) = 1.680, p = 0.102, resp.). No group differences were found for sleep latency (*F*(1,32) = 0.286, p = 0.597), REM latency (*F*(1,32) = 1.068, p = 0.309), REM density (*F*(1,32) = 0.555, p = 0.462), number of REM periods (*F*(1,32) = 0.125, p = 0.723), stage N1% (*F*(1,32) = 0.042, p = 0.838), stage N2% (*F*(1,32) = 1.226, p = 0.276), stage N3% (*F*(1,32) = 0.742, p = 0.395) ) and the amount of periodic leg movements with (*t* = −1.335, p = 0.201) or without arousals (*t* = −1.228, p = 0.237).

**Table 3:** **Sleep parameters of the three study nights for nightmare sufferers (NM) and healthy controls (HC)**

**Variable**	**Night 1**		**Night 2**		**Night 3**	
	**NM**	**HC**	**NM**	**HC**	**NM**	**HC**
Sleep period time (min)	453.73 ± 59.57	453.35 ± 68.57	454.70 ± 48.86	462.55 ± 72.73	430.11 ± 42.16	464.35 ± 54.12
Sleep efficiency (%)	83.51 ± 9.19	84.99 ± 7.06	90.99 ± 3.23	88.21 ± 7.08	90.28 ± 3.82	90.47 ± 4.25
Sleep onset latency (min)	18.76 ± 13.51	22.50 ± 23.87	15.26 ± 9.55	18.85 ± 16.90	16.97 ± 13.25	16.82 ± 11.46
REM latency (min)	105.47 ± 50.44	104.32 ± 48.36	78.79 ± 37.48	94.50 ± 40.95	81.47 ± 31.87	99.88 ± 43.70
REM density	20.78 ± 6.45	18.86 ± 5.44	21.06 ± 5.07	20.52 ± 5.71	21.11 ± 6.90	19.50 ± 6.41
Number of awakenings	26.94 ± 10.07	23.82 ± 8.79	24.82 ± 9.36	24.59 ± 10.89	22.41 ± 6.22	26.35 ± 9.17
Number of REM periods	3,06 ± 0,899	2,94 ± 0,966	3,82 ± 0,636	3,53 ± 1,419	3,29 ± 1,213	3,41 ± 1,004
Arousal Index	10.68 ± 3.64	8.78 ± 1.86	10.38 ± 3.35	9.09 ± 2.04	9.33 ± 2.62	9.75 ± 2.06
Awake%	11.45 ± 7.58	9.54 ± 5.33	5.67 ± 2.88	6.48 ± 5.05	5.76 ± 2.72	5.82 ± 3.59
Stage N1%	9.22 ± 3.03	8.03 ± 3.91	8.33 ± 3.92	8.20 ± 3.96	8.39 ± 3.93	9.00 ± 2.94
Stage N2%	42.74 ± 5.92	46.41 ± 6.18	45.25 ± 7.62	46.50 ± 9.01	45.01 ± 6.77	47.35 ± 7.11
Stage N3%	22.48 ± 9.24	20.77 ± 11.04	23.96 ± 9.30	20.95 ± 9.68	24.75 ± 7.87	21.57 ± 9.52
Stage REM%	13.92 ± 3.60	14.70 ± 3.85	16.75 ± 3.84	15.41 ± 4.21	16.05 ± 3.68	16.20 ± 3.70
Arousal Index (REM only)	9.85 ± 5.05	7.57 ± 3.47	9.66 ± 4.77	9.20 ± 4.43	10.37 ± 5.24	8.15 ± 4.23
Periodic leg movements	3.00 ± 6.36	1.95 ± 3.81				
Periodic leg movements with arousal	0.18 ± .59	0.23 ± .50				

Results are presented as mean ± standard deviation.

We found a marginally significant Group × Night interaction for the number of nocturnal awakenings (*F*(2,64) = 2.894, p = 0.063, η_p_^2^ = 0.083) with decreasing awakenings in the NM group and increasing awakenings in the HC group. Moreover, there was a significant Group × Night interaction for AI (*F*(2,64 = 4.023, p = 0.023, η_p_^2^ = 0.112) represented by decreasing AI in the NM group and increasing AI in the HC participants. No other interactions were found.

Because no group differences could be found in sleep architecture over all three nights, we further analyzed the data by comparing only those nights where nightmares occurred in the NM group. For comparison, nights of the HC participants were selected by matching age, night and time of nightmare with those dreams of the NM group. Results are presented in Table 4. Again, no significant group differences could be found.

**Table 4:** **Sleep parameters of nights with nightmares in frequent nightmare sufferers (NM) compared with matched nights of healthy controls (HC)**

**Variable**	**NM**	**HC**	**NM vs. HC**		**Effect size**
	**n = 11**	**n = 11**	***t***	**p**	**d**
Sleep period time (min)	444.63 ± 58.20	461.36 ± 61.98	−0.652	0.522	0.28
Sleep efficiency (%)	84.73 ± 9.65	85.65 ± 6.64	−0.262	0.796	0.11
Sleep onset latency (min)	20.77 ± 13.55	15.364 ± 10.02	1.064	0.300	0.45
REM latency (min)	86.54 ± 42.78	95.04 ± 39.64	−0.483	0.634	0.21
REM density	21.68 ± 8.10	22.80 ± 7.00	−0.348	0.731	0.15
Number of awakenings	28.36 ± 11.38	28.73 ± 8.90	−0.083	0.467*	0.04
Number of REM periods	3.45 ± 1.29	3.27 ± 0.64	0.417	0.683	0.18
Arousal Index	10.63 ± 3.19	9.46 ± 1.21	1.130	0.140*	0.48
Awake%	11.02 ± 9.06	10.95 ± 6.07	0.022	0.983	0.01
Stage N1%	10.14 ± 3.19	8.24 ± 3.36	1.353	0.191	0.58
Stage N2%	44.89 ± 6.61	44.23 ± 7.02	0.227	0.823	0.10
Stage N3%	19.83 ± 6.86	21.22 ± 8.51	−0.423	0.677	0.18
Stage REM%	14.08 ± 3.85	15.32 ± 3.53	−0.786	0.441	0.34
Arousal index (REM only)	10.73 ± 5.15	12.09 ± 8.38	0.519	0.305*	0.22
Periodic leg movements	1.78 ± 5.91	1.18 ± 3.94	0.278	0.784	0.12

Results are presented as mean ± standard deviation.

*One-tailed t-test.

We further looked at the distinct sleep cycles to potentially find differences in sleep microstructure. First, the number of REM periods in each night will be presented for NM and HC participants. *Night 1, NM subjects*: 5 NMs with two REM periods, 7 NMs with three REM periods, 4 NMs with four REM periods and 1 NMs with five REM periods. *Night 1, HC subjects*: 1 HCs with one REM period, 4 HCs with two REM periods, 8 HCs with three REM periods, 3 HCs with four REM periods and 1 HCs with five REM periods. *Night 2, NM subjects*: 5 NMs with three REM periods, 10 NMs with four REM periods and 2 NMs with five REM periods. *Night 2, HC subjects*: 1 HCs with one REM period, 3 HCs with two REM periods, 4 HCs with three REM periods, 6 HCs with four REM periods, 2 HCs with five REM periods and 1 HCs with seven REM periods. *Night 3, NM subjects*: 5 NMs with two REM periods, 6 NMs with three REM periods, 3 NMs with four REM periods, 2 NMs with five REM periods and 1 NMs with six REM periods *Night 3, HC subjects*: 3 HCs with two REM periods, 7 HCs with three REM periods, 4 HCs with four REM periods and 3 HCs with five REM periods.

In the next step, repeated measure comparisons of the first, second, third and fourth REM/NREM cycles were conducted. Results are presented in Table 5. Again, no group differences could be found for cycle length (*F*(1,32) = 0.553, p = 0.463; *F*(1,30) = 0.478, p = 0.494; *F*(1,15) = 0.055, p = 0.818; *F*(1,5) = 0.033, p = 0.863), duration of REM in the respective cycle (*F*(1,32) = 0.006, p = 0.941; *F*(1,32) = 0.301, p = 0.587; *F*(1,30) = 0.202, p = 0.657; *F*(1,15) = 1.104, p = 0.310) or REM density (*F*(1,32) = 0.351, p = 0.558; *F*(1,32) = 0.034, p = 0.855; *F*(1,30) = 0.064, p = 0.802; *F*(1,15) = 1.455, p = 0.246). No Night effects or Group × Night interactions could be found.

**Table 5:** **REM sleep parameters by different sleep cycles and nights for frequent nightmare sufferers (NM) and healthy controls (HC)**

**Cycle**	**Variable**	**Night 1**		**Night 2**		**Night 3**	
		**NM**	**HC**	**NM**	**HC**	**NM**	**HC**
1	Cycle length	103.47 ± 32.06	104.70 ± 19.61	94.05 ± 18.67	110.08 ± 51.54	100.26 ± 21.10	97.94 ± 18.08
	REM duration	13.08 ± 13.85	13.94 ± 7.96	13.94 ± 10.98	13.52 ± 10.98	14.02 ± 6.91	14.11 ± 9.48
	REM density	15.13 ± 6.76	13.57 ± 6.65	14.76 ± 7.20	13.73 ± 8.53	13.57 ± 6.30	13.24 ± 6.30
2	Cycle length	106.64 ± 26.22	96.30 ± 22.13	93.05 ± 27.12	90.39 ± 25.86	99.67 ± 12.02	101.36 ± 16.59
	REM duration	24.35 ± 15.42	20.94 ± 10.55	15.29 ± 9.25	21.76 ± 10.73	25.44 ± 8.78	25.58 ± 9.70
	REM density	20.23 ± 8.72	18.90 ± 8.33	17.35 ± 9.53	19.56 ± 9.60	21.48 ± 10.6	19.21 ± 10.49
3	Cycle length	92.49 ± 22.09	88.00 ± 37.16	80.72 ± 22.00	83.75 ± 21.22	80.77 ± 15.93	77.56 ± 17.62
	REM duration	20.58 ± 11.60	21.53 ± 8.01	23.35 ± 10.27	18.96 ± 15.31	24.26 ± 13.71	23.93 ± 12.21
	REM density	19.77 ± 9.57	21.65 ± 7.76	21.22 ± 8.60	21.25 ± 12.29	18.83 ± 6.44	18.37 ± 5.92
4	Cycle length	83.00 ± 10.10	79.83 ± 13.96	77.37 ± 9.31	84.16 ± 8.37	72.62 ± 8.26	66.16 ± 25.15
	REM duration	28.00 ± 14.77	32.18 ± 8.92	22.61 ± 15.64	27.06 ± 11.14	21.11 ± 10.25	23.12 ± 12.69
	REM density	21.15 ± 8.95	23.26 ± 9.01	21.52 ± 6.59	24.62 ± 8.94	22.46 ± 13.31	30.82 ± 9.35

Results are presented as mean ± standard deviation; cycle length and REM duration are presented in minutes.

## Discussion

Among the few studies designed to examine the effects of nightmares in multiple modalities, this is the first to do so under ecologically valid conditions. Our findings support the notion that sleep in people with frequent nightmares is subjectively disturbed compared to healthy controls [20,26,31]. Individuals who suffer from nightmares reported sleep problems (e. g., impaired sleep quality and insomnia complaints) as well as daytime dysfunction like tiredness and mood problems. However, these complaints could not be confirmed by ambulatory polysomnographic recordings. We found no differences between both groups regarding the sleep parameters when (a) total sleep over all three night was considered, (b) sleep cycles were analyzed regarding cycle length, amount of REM sleep and REM density and (c) only the nights with nightmares in the NM group were compared with corresponding nights of control participants (without nightmares).

Although no differences in sleep patterns could be found when only nights with nightmares and comparable nights of control participants with neutral dreams were analyzed, some results have to be discussed more detailed. Highest effect sizes (small-medium to medium) were found for sleep onset latency (NM longer), arousal index (NM higher), percentage of stage N1 (NM more) and percentage of stage REM (NM less). All differences point in the expected direction which suggests that nightmare sufferers may potentially differ in sleep architecture. Overall, we found almost exclusively high effects on self reported sleep quality with significant differences between both groups and some small to medium effects for objective sleep quality that were not significantly different. Nightmares seem to influence subjective sleep quality more severely than physiological sleep pattern. Future ambulatory studies should include more NM subjects to unveil possible sleep pattern differences.

Our finding of comparable sleep architecture between nightmare sufferers and healthy controls is contrary to two prior studies showing that there are differences in sleep patterns [31,32]. It is possible that nightmare sufferers are more sensitive to changes in their usual sleep practice and therefore, exhibit worse and more aroused sleep in laboratory settings. This assumption is supported by a recent study which has shown an enhanced first-night effect in NM subjects [47]. The first-night effect refers to the fact that sleep differs between the first and second night in sleep laboratory settings. The authors underline that their finding emerged mainly due to the large baseline difference: NM subjects slept much worse during the first night [47]. An advantage of the present investigation is the ambulatory setting for polysomnographic recordings. Applying this procedure, we found no differences in the sleep architecture between frequent nightmare sufferers and healthy controls. Moreover, prior research suggests that nightmares are less frequent in laboratory settings [48]. It is possible that differences in sleep architecture in the laboratory studies are due to the unnatural sleep environment [49]. More ambulatory studies are needed to further clarify this assumption.

Another explanation for the contrary findings could be that all studies which investigated differences in sleep architecture had more or less different designs, procedures and ways of analyzing the data. Both studies that found differences analyzed sleep parameters of the second of two nights only, the first night served as an adaptation night [31,32]. Those two studies that found no differences [33,34] investigated the effects of REM sleep deprivation (night one and three were analyzed; in the second night, participants were deprived of REM sleep). Therefore, the results should be treated with caution because sleep architecture is affected by REM deprivation and findings from the first night could be biased by first-night effect [47]. These differences in the designs of previous polysomnographic nightmare studies could explain the different findings and limit general conclusions.

Furthermore, characteristics of the control participants could also influence the results. Our controls scored consistently higher on almost all measures of sleep quality and there were no signs of either mental or sleep disorders. They were included if nightmares occurred less than once a month. Two studies had the same criteria [33,34], one included controls with less than two nightmares/bad dreams during the last year [31] and the other one did not specify this issue [32]. However, other studies may have included different controls, none of the previous investigations [31-34] found differences in terms of sleep efficiency for example.

It is possible that the results of previous studies were influenced by different characteristics of nightmare sufferers. In one investigation which found differences in sleep patterns between NM and control participants [32], depression and anxiety measures were not able to distinguish between both groups. The second study that found differences [31] included NM participants with higher scores for self reported symptoms of depression and anxiety than control subjects. However, in those two studies where no differences in sleep architecture were observed [33,34], nightmare sufferers also reported higher scores in anxiety and depression symptoms. In the present study groups did not differ in terms of depression symptoms. Based on these inconclusive findings, it can not be assumed that variations in the characteristics of the nightmare sufferers leaded to the different results.

We found that the number of REM periods and the amount of REM sleep increased over the nights, while the time awake after sleep onset decreased. The other sleep stages did not differ in the course of the investigation. It seems that with decreasing time awake, the amount of REM sleep increased together with the number of REM periods. This could be explained by REM rebound. Maybe our participants did not get their adequate amount of REM sleep in the first night which leads to increased REM pressure and therefore, more REM sleep and periods in the following night(s). One could also think about the first-night effect to explain the pattern but our data does not allow final conclusions.

Expectation anxiety in nightmare sufferers could also explain differences between subjective and objective sleep quality. The fear of falling asleep and having severe nightmares affect subjective sleep quality but may not influence physiological sleep patterns. Future studies should evaluate the magnitude of expectation anxiety before each investigated night. Previous research also revealed a connection between subjective stress and nightmare distress as well as psychopathology and nightmare distress [28]. As we did not ask questions regarding nightmare distress, it would be interesting to conduct ambulatory studies differentiating between nightmare frequency and nightmare distress.

Some limitations of our findings have to be addressed. Our participants had to write dream reports each time they woke up in the night and remembered a dream. This leads to artificially prolonged waking periods and may mask differences in comparison to undisturbed sleep patterns, e. g. sleep efficiency. It is possible for example, that people without frequent nightmares fall asleep much faster after awakening from a dream than nightmare sufferers do. Therefore, future studies should include control groups or nights in which no dream reports have to be provided.

Even a prior study [22] reported that the relationship between nightmare frequency and poor sleep quality was still significant after controlling neuroticism scores, it would be very interesting to study whether neuroticism as a trait (not measured in the present study) is related to subjective and objective sleep parameters.

## Conclusions

Our results suggest that nightmare disorder is independent from objective sleep disturbances but causes waking distress, negative mood, non-restorative sleep and tiredness during the day. Therefore, nightmares should be seen as a distinct sleep disorder with specific symptoms that needs specific treatment. Future studies should concentrate on ambulatory polysomnographic recordings to investigate sleep in natural environments to detect sleep and dream complaints in people with nightmares. Furthermore, the difference between nightmare distress and nightmare frequency should be regarded which may lead to new insights concerning objective sleep measures.

## Abbreviations

AI: Arousal index
AIR: Arousal index in REM sleep only
BDI: Beck depression inventory
BPD: Borderline personality disorder
ECG: Electrocardiogram
EEG: Electroencephalogram
EMG: Electromyogram
EOG: Electrooculogram
ESS: Epworth sleepiness scale
HC: Healthycontrols
LISST: Landecker Inventar zur Erfassung von Schlafstörungen
N1%: Percentage of light sleep stage 1 after sleep onset
N2%: Percentage of normal sleep stage 2 after sleep onset
N3%: Percentage of slow wave sleep stage 3 after sleep onset
NM: Nightmare sufferers
PSG: Polysomnography
PTSD: Posttraumatic stress disorder
R%: Percentage of REM sleep after sleep onset
REM: Rapid eye movement
SEI: Sleep efficiency index
SF-A: Schlaffragebogen A
SF-B: Schlaffragebogen B
SPT: Sleep period time
W%: Percentage of wakefulness after sleep onset
